# Supplementary material for: Weighted gene co-expression network analysis identifies functional modules related to bovine respiratory disease
Source: PLoS One. 2025 Oct 27;20(10):e0334688. doi: 10.1371/journal.pone.0334688 (PMC12558457; doi:10.1371/journal.pone.0334688)
Supplement: Attachment — Submitted filename: Review_Report_PONE-D-25-08222.docx [file pone.0334688.s001.docx]

**Summary:**

This article performed a meta-analysis on sequencing data from several articles where cattle were diagnosed or challenged with pathogens related to bovine respiratory disease (BRD). The article identified several differentially expressed genes, transcription factors, and enriched pathways associated with BRD.

**Overall Comments:**

- The authors should consult a native English speaker to improve the readability of this article.
- The authors have a well-organized methods section but should be sure to include sufficient detail for the methods being described. The authors should describe how they searched for datasets to include in their meta-analysis and what inclusion/exclusion criteria were applied. Additionally, the authors should justify their methodological choices (e.g., line 178: removing samples with a standard connectivity score less than -2.5).
- The overall structure/organization of the discussion had logical flow. Within each paragraph of the discussion, I encourage the authors to condense and summarize the information rather than discussing each point individually. Coalescing the relevant information into a single overarching statement with better contextualizing the relevance of each outcome on BRD would improve the readability of the discussion.
- In general the authors should familiarize themselves with current research on biomarkers in animal health and BRD to be able to more appropriately interpret their findings.

**Specific Comments:**

**Abstract:**

- Line 12: The phrase pathogenic pathogens is misleading, many organisms involved in BRD are commensals.
- Line 27: Please revise, CFB was identified as a potential biomarker but remains unvalidated. The authors should also provide more supporting evidence for the identification of CFB as a potential biomarker.
- Line 28-29: The authors should revise their conclusion to not interpret the findings of this study. Moreover, the authors do not provide sufficient evidence or for readers to interpret how these results are relevant to a producers breeding program.

**Introduction:**

- Consider restructuring the introduction for a more organized flow of information that identifies the authors’ key knowledge gap that they are trying to address with this article.
- The authors should prioritize the information they included in the introduction. There are instances where the authors state information that feels insufficiently contextualized to be relevant for readers (e.g., Line 52, Line 53-55).
- Line 33: Please expand on the complexities associated with the host immune system with regards to BRD.
- Line 34-36: Consider rephrasing for better flow. Please expand on what non-immunological defense mechanisms is the author referring to.
- Line 37-40: Are these the only pathogens associated with BRD, are all of these considered primary causes of BRD, please elaborate.
- Line 40-42: Consider moving the economic importance of BRD earlier in the introduction. The phrase “identification of key genes” feels out of place in this sentence. The other preventative and controls measures listed are major mechanisms or reducing producer losses associated with BRD. Without additional information with regards to how “identification of key genes” serves as a major control measure for BRD readers are caught off guard by the suggestion.
- Line 42-43: Consider rephrasing so the aim is more specific.
- Line 44-48: A nice description of RNA-Seq technology.
- Line 48-49: Could the authors elaborate about what is meant by “systematic approaches”.
- Line 49-50: Consider rephrasing this sentence, it makes it seem as though the aim you proposed has already been tested and that the work you are proposing has already been performed.
- Line 51: Please revise this sentence. The citations used to support this statement are inappropriate. According to the FDA a biomarker is a defined as a characteristic that is measured as an indicator of normal biological processes, pathogenic processes, or responses to an exposure or intervention, including therapeutic intervention ([see link](https://www.fda.gov/drugs/biomarker-qualification-program/about-biomarkers-and-qualification#:~:text=The%20Biomarkers%2C%20EndpointS%20and%20other,or%20intervention%2C%20including%20therapeutic%20interventions.)). The authors should be cautious not to overstate the utility of biomarkers or need to add clarifying details about how they expect producers utilize biomarkers to enhance their genetic programs.
- Line 52: Please revise this sentence. The authors have not provided sufficient background for readers to contextualize the importance of these genes and their relevance to this article. The authors should provide more background about the importance/role of these genes and why they are relevant to the author’s study.
- Line 53-55: Please revise this sentence. The authors have not provided sufficient background for readers to contextualize the importance of these genes and their relevance to this article beyond the fact that they have been identified as being relevant for an immunological response. The authors should provide more background about the importance/role of these genes and why they are relevant to the author’s study.
- Line 56: The authors should provide space to more clearly distinguish between the paragraphs (similarly to how they do it in the discussion)
- Line 56-57: This citation is inadequate to support this statement. One example of a study that was underpowered is not sufficient for supporting a claim that individual research as a whole is restricted on sample size, statistical power, and accuracy of outcomes. Moreover, this reference was not related to a BRD RNA-seq study or even performed in cattle leading readers to question how applicable that statement is to the proposed area of study. Furthermore, the sentence is redundant as sample size and power highly related based on the outcome of interest.
- Line 57-58: The authors should be more specific regarding how different platforms can lead to genotypic variation and elaborate on how meta-analysis addresses these issues?
- Line 60: Please elaborate on how this study is relevant to the work the authors performed.
- Line 61: Is this the start of a new paragraph? If so please correct the formatting to make it more clear.
- Line 66: Please clarify the relevance of tumor expression profiles to the author’s BRD meta-analysis
- Line 67-69: Please include citations to support this statement.
- Line 71: The term patients implies that the non-healthy samples were collected in a clinical setting.

**Methods:**

- The authors should improve the reporting about individual studies being used in the meta-analysis.

*Data Collection*

- Line 77-79: Could the authors please state what keywords were used to identify articles and what inclusion/exclusion criteria were applied to obtain the studies used in this meta-analysis.
- Line 80: Could the authors clarify what method of disease identification was used.
- Line 87-88: Please reword, rarely are viral pathogens considered primary causes of BRD (Taylor JD, Fulton RW, Lehenbauer TW, Step DL, Confer AW. The epidemiology of bovine respiratory disease: What is the evidence for predisposing factors? Can Vet J. 2010 Oct;51(10):1095-102. PMID: 21197200; PMCID: PMC2942046.)

*Supervised Machine-Learning Models*

- Could the authors elaborate on their choice to use supervised machine-learning instead of unsupervised machine learning.
- What data sets were used to perform the 10-fold cross validation to test the model’s accuracy.

**Results:**

- Line 176-178: Consider moving this discussion about outlier removal to the methods section. Moreover, please describe what criteria was used to identify outliers and how they were selected for removal.
- Line 229-239: Consider rewording this section to improve flow and readability, it is difficult for readers to follow how CFB is classified as a biomarker when CFB expression greater than 131.5 is associated with BRD (line 234/235), but later CFB expression greater than 131.5 was also healthy (line 236/237).

**Figures/tables:**

- For all figures, in addition to figure titles could the authors provide a detailed figure legend that describes the figure in sufficient detail that a reader can understand and interpret the information being presented without referencing the article. Be sure to include information such as sample size, statistical methods used, and interpretation of the figure.
- Table 1: Consider expanding this table to include more demographic data from each of the studies (e.g., sex, pathogens challenged with or identified, challenge vs. natural infection) and consider replacing specie with breed given that all samples were from *Bos taurus* cattle this column is not very informative; however, information about breed could be informative to readers. Moreover, the authors should include more information about the type of sequence performed by each of the article (depth of coverage, machine used, paired end reads, long read vs short read vs sanger sequencing, etc.).
- Figure 7: Please correct the typo “tress” to “trees”. Additionally, in the figure legend please provide sufficient context for readers to be able to interpret the figure.

**Discussion:**

- Throughout the discussion the authors make several statements that lack the background information necessary for readers to understand how they are relevant to the study or their importance in BRD.
  - One example is line 271, where the authors mention that NOD2 is a polymorphism related to viral pathogenesis, but fail to state what the polymorphism is or is it the entire gene that encodes for NOD2, they also fail to elaborate on why this is important for viral pathogenesis, is it viral pathogenesis of all viruses or specific viruses are any of the viruses relevant to BRD.
- The disorganization of the discussion and failure to discuss results within the context of bovine respiratory disease precludes readers ability to draw meaningful conclusions from this study.
- The authors should include a limitations section. The authors should include, but are not limited to, a discussion about how the use of animals who were naturally infected versus challenged with BRD pathogens may impact results and a discussion of any limitations in the author’s methods.
- Line 242: Please revise the sentence, the phrase “intricate multiple genes may correspond to” is difficult for readers to understand the author’s intended meaning
- Line 243-244: Please revise, this is the first time the authors have talked about BRD treatment. They should either provide more background information and contextualization or remove the sentence as it disrupts the flow of the discussion and is poorly supported.
- Line 244: Please elaborate on how understanding the underlying mechanisms of a disease will specifically help with the identification of animals vulnerable to BRD.
- Line 245-247: The authors never perform a power analysis to justify the appropriate number of studies/n to include to in their meta-analysis to achieve appropriate statistical power. Nor do the authors discuss if any of the articles presented were actually under powered.
- Line 250: The authors should justify their choice of supervised vs. unsupervised machine learning here or in the methods section.
- Line 252: The authors should justify their choice of using Fischer and Invorm methods for meta-gene identification here or in the method section.
- Line 258-314: This section needs major revisions. There are several instances where the authors reference the role of a gene relative to systems completely unrelated to cattle, BRD, or respiratory disease (e.g, line 264 liver cancer, line 266 cancer cell lines, 273 bowel disease and breast cancer, line 298 Epstein-Barr virus transcytosis, line 299 chagas disease, line 313 endometriosis, line 314 autoimmune disease, etc.). The authors should revise this section and focus on discussing and interpreting their findings within the context of bovine respiratory disease complex since that is the model they used.
- Line 277 – 278: Please clarify the relationship between regulating immunological functions and how that specifically “effectively protects” cells against oxidative damage.
- Line 299-301: Could the authors please clarify what important function Epstein-Barr virus transcytosis has in triggering inflammatory genes during BRD. Moreover could the authors clarify that same information for Chagas disease and mitophagy as well.
- Line 315-316: Could the authors please be more specific and clarify what genetic and non-genetic factors are relevant to BRD and provide more context for why the authors believe that there should be some genes that regulate the disease process.
- Line 317-319: Could the authors clarify why the ML algorithms were suitable for these predictions.
- Line 320: That authors should be cautious not to overinterpret results, stating that some animals were highly susceptible to BRD when some of the studies analyzed were challenge studies where disease was intentionally induced in animals is misleading to readers.
- Line 325-326: Please clarify the relevance to BRD.
- Line 326: Mastitis disease is redundant wording.
- Line 326-327: This sentence is disruptive to the flow of the discussion and does not provide any meaningful contextualization or aid in the interpretation of the results of the authors study, consider removing.
- Line 329-330: Please elaborate on this statement. As written it is not clear why NLRC5 can be considered a novel BRD gene.
- Line 334-335: Please elaborate on the relevance of this to BRD.
- Line 337: Consider connecting this back to BRD.
- Line 338-339: Please elaborate on the relevance of this to BRD.
- Line 340: Please be more specific.

**Conclusions:**

- The authors do a good job not overstating most of their findings in the conclusion and highlighting the need for additional research for biomarker discovery.
- Line 346 – 348: Please reword to improve readability. The authors only analyzed RNA-seq and therefore only identified genes that may be of interest. The authors can discuss the relevance of gene clustering with regards to molecular functions immune cell functions, but should be cautious not to over interpret the findings here.
