# Supplementary material for: Weighted gene co-expression network analysis identifies functional modules related to bovine respiratory disease
Source: PLoS One. 2025 Oct 27;20(10):e0334688. doi: 10.1371/journal.pone.0334688 (PMC12558457; doi:10.1371/journal.pone.0334688)
Supplement: Attachment — Submitted filename: Response to Reviewers.docx [file pone.0334688.s002.docx]

**Reviewer #1: •**

1- Line 12 (abstract): The authors highlight their use of a "P-value combination approach" regarding a brief summarization of their methods for the abstract. This is somewhat ambiguous, as their methodology in a combinational approach of WCGNA, machine learning workflows, and statistical methods (ex: DESeq2). The lack of specificity in this sentence may distract future readers finding their abstract in literature searches; they may consider removing this part of the sentence and instead emphasize the aspect of the meta-analysis approach (including the number of studies included).

Dear reviewer, Thank you for your insightful consideration of our manuscript. in current study, comprehensive meta-analysis was performed using P-value combination approach, in the next step, the identified meta genes were subjected the systems biology analysis using weighted gene co-expression network method. Then, the most functionally importance modules and genes were further validated using machine-learning approach. Finally, the critical regulatory network associated with BRD were constructed.

2- Line 39-42 (and throughout): minor typographical errors and run-on sentences were identified. For example, it is believed that "through" should be "thorough". Additionally, it is unclear how the "identification of key genes" would prevent and control BRD; it is believed that the authors are eluding to how these identifications would lead to the development/employment of early disease detection systems.

Dear reviewer, Thank you for your insightful consideration of our manuscript. has been extensively investigated (3, 4). Detecting key genes involved in *BRD*, enables a better understanding of the biological pathways that contribute to disease resistance, particularly those related to immune response and inflammation (5, 6).

3- Line 78-79: it is unclear why the investigators specifically elected to use these five studies. There are additional published studies that have used whole blood as the tissue sample of evaluation (assumed as the connecting theme of these works), but the authors should expand upon the rationale behind these studies. The utility and approach behind performing a meta-analysis-like study in bioinformatics remains a highly contested subject, so it is assumed there are no strict criteria as to how to perform a study of this type. However, the authors should provide context and rationale when possible as this work may be evaluated in the future as a way of performing this type of study (outside of BRD or animal health research).

Dear reviewer, Thank you for your insightful consideration of our manuscript. these studies were selected based on several key criteria to ensure the reliability and reproducibility of the results. Only studies that utilized whole blood as the biological sample were included, as this provides consistency in tissue-specific gene expression profiles and minimizes variability introduced by different tissue types. This allows for independent verification, re-analysis, and integration with other datasets as part of meta-analytical approaches. Table 1, citation 20: this citation should be revised; DOI: 10.1016/j.ygeno.2020.07.014. It was revised, please see the citations in the Table 1

4- Lines 95-118, methods: the authors utilized previously published RNA-Seq data for their statistical and combinational (p-value) analyses. However, I would express concern regarding the possibility of batch effects/technical bias across these five studies, as the laboratory techniques and bioinformatic processing, including alignment tools and reference assembly, are different across these projects (ex. Sun et al., 2020 utilized TopHat2 and the UMD3.1 genome, Johnston et al., 2021 utilized STAR and the UMD3.1 genome, and the work from the Jiminez, O’Donoghuem and Li publications utilized STAR and the UCD1.2 genome). The way this section is currently written, it is assumed the investigators acquired and combined the raw gene counts from each of these publications (not explicitly stated). The authors are recommending to comment and elaborate on their approach, as without the re-processing of combined raw data nor the use of a batch effect adjustment of raw gene counts (see DOI: 10.1093/nargab/lqaa078), this project risks presenting confounded results.

Dear reviewer, Thank you for your insightful consideration of our manuscript. to minimize the impact of batch effects resulting from variations in experimental protocols, sequencing platforms, alignment tools, and reference genome annotations, we first used a normalization approach implemented in the DESeq2 software. This method effectively reduces unwanted sources of variation commonly encountered in high-throughput sequencing experiments, thereby enhancing the accuracy and comparability of gene expression measurements.

Lines 100-102: which testing procedure was used with DESeq2 (i.e., Wald testing, likelihood ratio testing, etc.)?

Dear reviewer, Thank you for your insightful consideration of our manuscript. The Wald test was calculated using *DESeq2* to assess the statistical significance of *DEGs* between conditions.

5- Line 125: the use of Pearson correlation coefficients with WGCNA (and generally within statistics) are often considered to be limited in interpretation due to the sensitivity to outlier data and risk of estimator bias, especially when applied to data with limited sample size. While the authors of WGCNA have often recommended to have a minimum of 15 samples (well surpassed by this study), they also recommend a more robust coefficient (such as the biweight midcorrelation) and signed networking for adjacency matrix construction. While this technique has been used and published previously, the investigators may comment on the rationale of this aspect of their methods.

# Dear reviewer, thank you for your critical reviewing of our manuscript. As you mentioned to increase the reliability of constructed co expression network, we performed the outlier detection step to remove the outliers from the study, moreover, after the removing the outliers the applied samples we higher than 15 samples, which increase the reliability of our study, furthermore, the applied approach were signed method for network construction.

6- Lines 130-142: which platform/software/toolkit was used to perform functional enrichment analyses? The authors explain the databases used, but not the specific toolkit (ex. DAVID, WebGestalt, gProfiler, etc.).

Dear reviewer, Thank you for your insightful consideration of our manuscript. To investigate the functional significance of the identified modules, enrichment analysis was carried out based on Gene Ontology *(GO)* and Kyoto Encyclopedia of Genes and Genomes (KEGG) using the STRING and DAVID databases (39). This procedure detects biological-processes, molecular-functions, and cellular-components that are crucially over-represented. Detecting over-represented biological and molecular pathways donates valued comprehension of biological mechanisms in *BRD*.

7- Lines 154-163: similar to the previous comment, it is unclear what program was utilized here. Additionally, the techniques described are supervised algorithms which require training and cross-validation. Therefore, a confusion matrix should be produced from these algorithms which would have demonstrated the classification statistics (i.e., F1 scores, AUCs, balanced accuracies, recall, precision, etc.). It is assumed that the authors utilized the balanced accuracies as their determination for model selection, but this should be explicitly stated and the full statistics of all models should be provided as a table or supplemental file.

Dear reviewer, Thank you for your insightful consideration of our manuscript. Supervised machine learning involve training a model on a dataset that includes both input features (genes) and known outputs (healthy or disease status). The process involved in machine learning can be broken down into several key stages: data pre-processing, data splitting, model selection, model training, and model evaluation (41). We employed machine learning algorithms to identify and validate the most significant meta-genes associated with *BRD*, ensuring precise gene prioritization with optimal accuracy.

9- Line 231: the values used in the decision tree are not described. Are these adjusted genes count values or do they represent something else?

Dear reviewer, Thank you for your insightful consideration of our manuscript. the provided values are the normalized expression values of genes.

8- Figures: it may be the formatting within the PDF file, but please ensure that the figures are of high quality/resolution. Specifically, Figure 3 is blurred and the "Fishercomb" header is clipped from the image.

Dear reviewer, Thank you for your insightful consideration of our manuscript. we revised and increased the quality of figure. Please see the revision format.

**Reviewer #2: •** The authors should consult a native English speaker to improve the readability of this article.

Dear reviewer, Thank you for your insightful consideration of our manuscript. we focused on improving the readability and logical flow of the article.

• The authors have a well-organized methods section but should be sure to include sufficient detail for the methods being described. The authors should describe how they searched for datasets to include in their meta-analysis and what inclusion/exclusion criteria were applied. Additionally, the authors should justify their methodological choices (e.g., line 178: removing samples with a standard connectivity score less than -2.5).

Dear reviewer, thanks you for your insightful comments, we utilized p-value combination approaches for meta-analysis. as described in methods and material section. To minimize the impact of batch effects arising from variations in experimental protocols, sequencing platforms, alignment tools, and reference genome annotations, normalization and batch effect reduction approach were performed.

For WGCNA, Following the removal of outlier samples, the remaining high-quality data were subjected to weighted gene co-expression network analysis to explore the underlying gene expression patterns. The soft-thresholding power (β) was selected by systematically evaluating the scale-free topology fit index (R²) across a range of candidate powers. The optimal β was defined as the smallest power at which the network began to exhibit approximate scale-free properties (R² > 0.8). This selection process ensures that the constructed network reflects the expected biological architecture of gene co-expression, thereby improving the accuracy and interpretability of subsequent module detection and functional analysis. Moreover, the heat map illustrated the *TOM* values to visualize the degree of interconnectedness among genes within the network modules identified using the dynamic tree cutting algorithm **(Fig 4C).** Yellow and progressively red colors demonstrate the low and higher *TOM* values, respectively. The gene expression patterns within each module were condensed into a single representative vector, named the module eigengene, which corresponds to the first principal component derived from the module’s expression data matrix **(Fig 4D).**

• The overall structure/organization of the discussion had logical flow. Within each paragraph of the discussion, I encourage the authors to condense and summarize the information rather than discussing each point individually. Coalescing the relevant information into a single overarching statement with better contextualizing the relevance of each outcome on BRD would improve the readability of the discussion.
Dear reviewer, Thank you for your insightful consideration of our manuscript. It was revised. Please see the revision.

• In general the authors should familiarize themselves with current research on biomarkers in animal health and BRD to be able to more appropriately interpret their findings.

Dear reviewer, Thank you for your insightful consideration of our manuscript. the *CFB* gene, which regulates cellular senescence, was observed to be upregulated in this study, consistent with previous findings suggesting its role as an immune-related gene during *BRD* (27, 85, 86).

**Summary:**

This article performed a meta-analysis on sequencing data from several articles where cattle were diagnosed or challenged with pathogens related to bovine respiratory disease (BRD). The article identified several differentially expressed genes, transcription factors, and enriched pathways associated with BRD.

**Overall Comments:**

- The authors should consult a native English speaker to improve the readability of this article.
- The authors have a well-organized methods section but should be sure to include sufficient detail for the methods being described. The authors should describe how they searched for datasets to include in their meta-analysis and what inclusion/exclusion criteria were applied. Additionally, the authors should justify their methodological choices (e.g., line 178: removing samples with a standard connectivity score less than -2.5).
- The overall structure/organization of the discussion had logical flow. Within each paragraph of the discussion, I encourage the authors to condense and summarize the information rather than discussing each point individually. Coalescing the relevant information into a single overarching statement with better contextualizing the relevance of each outcome on BRD would improve the readability of the discussion.
- In general the authors should familiarize themselves with current research on biomarkers in animal health and BRD to be able to more appropriately interpret their findings.

**Specific Comments:**

**Abstract:**

- Line 12: The phrase pathogenic pathogens is misleading, many organisms involved in BRD are commensals.

Dear reviewer, we have removed the pathogenic pathogens as suggested.

- Line 27: Please revise, CFB was identified as a potential biomarker but remains unvalidated. The authors should also provide more supporting evidence for the identification of CFB as a potential biomarker.

Dear reviewer, Finally, the constructed model revealed that differential expression of the CFB gene was significantly associated with susceptibility to BRD. In cattle, CFB expression has been correlated with clinical signs of respiratory disease, supporting its role as a biomarker. Furthermore, the involvement of CFB in modulating pro-inflammatory cytokines (TNF-α) and its integration with other immune-related pathways (NF-κB signaling) adds to its relevance as a biomarker.

- Line 28-29: The authors should revise their conclusion to not interpret the findings of this study. Moreover, the authors do not provide sufficient evidence or for readers to interpret how these results are relevant to a producers breeding program.

Dear reviewer, Overall, this integrative approach significantly enhances our understanding of the molecular mechanisms underlying BRD and provides the groundwork for diagnostic, therapeutic, and genetic selection strategies aimed at improving cattle health and disease resistance.

**Introduction:**

- Consider restructuring the introduction for a more organized flow of information that identifies the authors’ key knowledge gap that they are trying to address with this article.
- The authors should prioritize the information they included in the introduction. There are instances where the authors state information that feels insufficiently contextualized to be relevant for readers (e.g., Line 52, Line 53-55).

Dear reviewer, the genes STAT3, IKBKG, IRAK1, NOD2, TLR2, TNFRSF1A, and IKBKB have been identified as key contributors to the immune response in cattle affected by respiratory disease.

And, a subset of RBD-related genes, including PLDA, PHLDA2, VNSC, and PAM, was predicted using a decision tree algorithm (DT) (1). This sentence was moved to another part of the introduction.

- Line 33: Please expand on the complexities associated with the host immune system with regards to BRD.

Dear reviewer, Bovine respiratory disease (BRD) is a multifactorial disease that involves intricate host-immune interactions influenced by environmental variables and pathogens (1). The innate immune system represents the first line of defense against BRD. Epithelial cells and immune sentinel cells prevent infection through secretion of proinflammatory cytokines. Neutrophils play an essential role in the pathogenesis of BRD by promoting inflammation and contributing to the destruction of lung tissue (2).

- Line 34-36: Consider rephrasing for better flow. Please expand on what non-immunological defense mechanisms is the author referring to.

Dear reviewer, Non-immunological defense mechanisms in BRD involve physical and chemical barriers, which act as a first line of defense before the adaptive immune system is engaged. These early defense systems play a vital role in limiting pathogen entry and controlling infection at the onset of disease.

- Line 37-40: Are these the only pathogens associated with BRD, are all of these considered primary causes of BRD, please elaborate.

Dear reviewer, these bacterial and viral pathogens represent some of the most prevalent and clinically impactful agents implicated in the development and progression of BRD.

- Line 40-42: Consider moving the economic importance of BRD earlier in the introduction. The phrase “identification of key genes” feels out of place in this sentence. The other preventative and controls measures listed are major mechanisms or reducing producer losses associated with BRD. Without additional information with regards to how “identification of key genes” serves as a major control measure for BRD readers are caught off guard by the suggestion.

Dear reviewer, Vaccination programs and treatment of subclinical animals are main approaches for the prevention and control of BRD. Additionally, identifying key genes associated with disease susceptibility can aid in developing targeted breeding programs and improving cattle resistance to BRD (7).

- Line 42-43: Consider rephrasing so the aim is more specific.

Dear Reviewer, we have revised sentences for improved clarity.

- Line 44-48: A nice description of RNA-Seq technology.

Dear Reviewer, RNA sequencing (RNA-Seq) is a powerful and comprehensive technique used to investigate gene expression profiles, functional mechanisms and molecular processes in distinct biological pathways (8, 9). RNA-Seq currently represents the most precise and reliable method for estimating gene expression levels (10).

- Line 48-49: Could the authors elaborate about what is meant by “systematic approaches”.

Dear Reviewer, we have revised sentences for improved clarity. Several RNA-Seq studies have successfully identified key genes and host molecular mechanisms involved in the pathogenesis of BRD (1, 11-13).

- Line 49-50: Consider rephrasing this sentence, it makes it seem as though the aim you proposed has already been tested and that the work you are proposing has already been performed.

Dear Reviewer, we have revised sentences for improved clarity. These studies have provided valuable insights into differential gene expression patterns, immune response pathways, and regulatory networks that contribute to disease susceptibility and progression (12, 14). The genes STAT3, IKBKG, IRAK1, NOD2, TLR2, TNFRSF1A, and IKBKB have been identified as key contributors to the immune response in bovine respiratory disease (15).

- Line 51: Please revise this sentence. The citations used to support this statement are inappropriate. According to the FDA a biomarker is a defined as a characteristic that is measured as an indicator of normal biological processes, pathogenic processes, or responses to an exposure or intervention, including therapeutic intervention ([see link](https://www.fda.gov/drugs/biomarker-qualification-program/about-biomarkers-and-qualification#:~:text=The%20Biomarkers%2C%20EndpointS%20and%20other,or%20intervention%2C%20including%20therapeutic%20interventions.)). The authors should be cautious not to overstate the utility of biomarkers or need to add clarifying details about how they expect producers utilize biomarkers to enhance their genetic programs.

Dear reviewer, we have removed and revised the sentences as suggested.

- Line 52: Please revise this sentence. The authors have not provided sufficient background for readers to contextualize the importance of these genes and their relevance to this article. The authors should provide more background about the importance/role of these genes and why they are relevant to the author’s study.

Dear reviewer, this sentence was moved to another part of the introduction.

- Line 53-55: Please revise this sentence. The authors have not provided sufficient background for readers to contextualize the importance of these genes and their relevance to this article beyond the fact that they have been identified as being relevant for an immunological response. The authors should provide more background about the importance/role of these genes and why they are relevant to the author’s study.

Dear reviewer, the genes STAT3, IKBKG, IRAK1, NOD2, TLR2, TNFRSF1A, and IKBKB have been identified as key contributors to the immune response in bovine respiratory disease (15).

- Line 56: The authors should provide space to more clearly distinguish between the paragraphs (similarly to how they do it in the discussion)

Line 56-57: This citation is inadequate to support this statement. One example of a study that was underpowered is not sufficient for supporting a claim that individual research as a whole is restricted on sample size, statistical power, and accuracy of outcomes. Moreover, this reference was not related to a BRD RNA-seq study or even performed in cattle leading readers to question how applicable that statement is to the proposed area of study. Furthermore, the sentence is redundant as sample size and power highly related based on the outcome of interest.

- Line 57-58: The authors should be more specific regarding how different platforms can lead to genotypic variation and elaborate on how meta-analysis addresses these issues?

Dear reviewer, line 56-58: Individual RNA-Seq studies often has limitations on sample sizes, platform variability, and statistical power (16). To address these challenges, meta-analysis serves as a robust and standardized approach for integrating data from multiple studies, thereby increasing statistical power and improving the reliability of gene expression findings (17). The meta-analysis method has been used to determine gene expression profiles and evaluate the relative effectiveness of antibiotics in controlling BRD in beef cattle (18).

- Line 60: Please elaborate on how this study is relevant to the work the authors performed.
- Line 61: Is this the start of a new paragraph? If so, please correct the formatting to make it more clear.
- Line 66: Please clarify the relevance of tumor expression profiles to the author’s BRD meta-analysis
- Line 67-69: Please include citations to support this statement.
- Line 71: The term patients implies that the non-healthy samples were collected in a clinical setting.

Dear reviewer, line 60-71: Weighted Gene Co-Expression Network Analysis (WGCNA) is a systems biology approach that is employed to identify modules of highly correlated genes (19). A co-expression network was constructed using WGCNA to determine the significant modules and candidate potential biomarkers in BRD group and healthy group (20).

**Methods:**

- The authors should improve the reporting about individual studies being used in the meta-analysis.

*Data Collection*

- Line 77-79: Could the authors please state what keywords were used to identify articles and what inclusion/exclusion criteria were applied to obtain the studies used in this meta-analysis.

Dear reviewer, we selected studies based on specific criteria, including the most recent research on BRD, availability of accessible count matrices, clearly defined case and control groups, sufficient sample sizes for robust statistical comparisons, and sequencing platforms.

- Line 80: Could the authors clarify what method of disease identification was used.

Dear reviewer, In the Pulled group, disease identification was based on a combination of clinical scoring, elevated rectal temperature, and veterinary diagnosis.

- Line 87-88: Please reword, rarely are viral pathogens considered primary causes of BRD (Taylor JD, Fulton RW, Lehenbauer TW, Step DL, Confer AW. The epidemiology of bovine respiratory disease: What is the evidence for predisposing factors? Can Vet J. 2010 Oct;51(10):1095-102. PMID: 21197200; PMCID: PMC2942046.)

Dear reviewer, we have revised the sentences as suggested. In the fourth dataset (GSE199108), the study included young Holstein-Friesian calves infected with Bovine Herpesvirus 1 (BoHV-1) (n = 12) and uninfected control calves (n = 6).

*Supervised Machine-Learning Models*

- Could the authors elaborate on their choice to use supervised machine-learning instead of unsupervised machine learning.
- What data sets were used to perform the 10-fold cross validation to test the model’s accuracy.

Dear reviewer, Supervised machine learning involves training a model on a dataset that includes both input features (genes) and known outputs (healthy or disease status). The process involved in machine learning can be broken down into several key stages: data pre-processing, data splitting, model selection, model training, and model evaluation (45). We employed machine learning algorithms to identify and validate the most significant meta-genes associated with BRD, ensuring precise gene prioritization with optimal accuracy. Feature selection algorithms based on weighting algorithms were applied to prioritize the identified key genes related to BRD. The hub genes in the significant modules are used to choose important features utilizing 10 distinct weighting algorithms, including information gain, information gain ratio, χ2, deviation, rule, support vector machine, Gini index, uncertainty, relief, and principal component analysis (PCA) (46). Next, the selected features were considered for the optimization of predictive modeling using Decision Trees (DTs) algorithm. To evaluate the model's accuracy, a 10-fold cross-validation approach was applied. In this method, the data were partitioned into 10 equal subsets, where 9 subsets were used for training and 1 subset for testing in each iteration to ensure that the selected model was thoroughly tested for generalizability and predictive performance. Ultimately, the model with the highest accuracy rate was proposed as the best predictor for identifying BRD-associated genes.

**Results:**

- Line 176-178: Consider moving this discussion about outlier removal to the methods section. Moreover, please describe what criteria was used to identify outliers and how they were selected for removal.

Dear reviewer, Following the removal of outlier samples, the remaining high-quality data were subjected to weighted gene co-expression network analysis to explore the underlying gene expression patterns. The soft-thresholding power (β) was selected by systematically evaluating the scale-free topology fit index (R²) across a range of candidate powers. The optimal β was defined as the smallest power at which the network began to exhibit approximate scale-free properties (R² > 0.8). This selection process ensures that the constructed network reflects the expected biological architecture of gene co-expression, thereby improving the accuracy and interpretability of subsequent module detection and functional analysis.

- Line 229-239: Consider rewording this section to improve flow and readability, it is difficult for readers to follow how CFB is classified as a biomarker when CFB expression greater than 131.5 is associated with BRD (line 234/235), but later CFB expression greater than 131.5 was also healthy (line 236/237).

Dear reviewer, the samples were diagnosed with BRD when the values of CFB and STAT2 genes were more than 131.500 and 9161, respectively. On the other hand, samples were identified as BRD when the expression values of when CFB expression was >131.5, STAT2 ≤9161, NLRC5 ≤11922, IL15RA ≤800, and ALPK1 >635. These findings indicate that CFB expression consistently appears in BRD classifications, depending on the expression context of other genes, such as STAT2, NLRC5, IL15RA, and ALPK1.

**Figures/tables:**

- For all figures, in addition to figure titles could the authors provide a detailed figure legend that describes the figure in sufficient detail that a reader can understand and interpret the information being presented without referencing the article. Be sure to include information such as sample size, statistical methods used, and interpretation of the figure.

Dear Reviewer, we have provided detailed explanations for all figures as requested.

- Table 1: Consider expanding this table to include more demographic data from each of the studies (e.g., sex, pathogens challenged with or identified, challenge vs. natural infection) and consider replacing specie with breed given that all samples were from *Bos taurus* cattle this column is not very informative; however, information about breed could be informative to readers. Moreover, the authors should include more information about the type of sequence performed by each of the article (depth of coverage, machine used, paired end reads, long read vs short read vs sanger sequencing, etc.).

Dear Reviewer, I was unable to incorporate all the points you mentioned into the manuscript. In one study, the breed of the animals used was clearly stated, but in the other studies, this information was not provided. The same limitation applied to several of the other aspects you noted as well.

- Figure 7: Please correct the typo “tress” to “trees”. Additionally, in the figure legend please provide sufficient context for readers to be able to interpret the figure.

Dear reviewer, we have revised the “tress” to “trees” as suggested. Additionally, we provided sufficient context to interpret the figure.

**Discussion:**

- Throughout the discussion the authors make several statements that lack the background information necessary for readers to understand how they are relevant to the study or their importance in BRD.
  - One example is line 271, where the authors mention that NOD2 is a polymorphism related to viral pathogenesis, but fail to state what the polymorphism is or is it the entire gene that encodes for NOD2, they also fail to elaborate on why this is important for viral pathogenesis, is it viral pathogenesis of all viruses or specific viruses are any of the viruses relevant to BRD.

Dear Reviewer, we have revised the sentence for improved clarity. Disruption of the NOD2 gene has been associated with impaired resistance to inflammatory diseases (59), while its interaction with BRSV suggests a potential role in the innate immune response to viral infection (60).

- The disorganization of the discussion and failure to discuss results within the context of bovine respiratory disease precludes readers ability to draw meaningful conclusions from this study.

Dear Reviewer, we have revised all sentences for improved clarity.

- The authors should include a limitations section. The authors should include, but are not limited to, a discussion about how the use of animals who were naturally infected versus challenged with BRD pathogens may impact results and a discussion of any limitations in the author’s methods.

Dear Reviewer, Additionally, including animals from naturally infected and experimental challenge studies may introduce variability in gene expression profiles.

- Line 242: Please revise the sentence, the phrase “intricate multiple genes may correspond to” is difficult for readers to understand the author’s intended meaning

Dear reviewer, intricate multiple genes convert to the complex regulation of these genes

- Line 243-244: Please revise, this is the first time the authors have talked about BRD treatment. They should either provide more background information and contextualization or remove the sentence as it disrupts the flow of the discussion and is poorly supported.

Dear reviewer, we have removed the sentence as suggested.

- Line 244: Please elaborate on how understanding the underlying mechanisms of a disease will specifically help with the identification of animals vulnerable to BRD.

Dear reviewer, we have removed the sentence as suggested.

- Line 245-247: The authors never perform a power analysis to justify the appropriate number of studies/n to include to in their meta-analysis to achieve appropriate statistical power. Nor do the authors discuss if any of the articles presented were actually under powered.

Dear reviewer, due to limited sample sizes in some individual studies, the statistical power for detecting reliable gene-level changes was insufficient. To overcome this limitation and enhance the reliability of results, a meta-analysis approach was employed.

Line 250: The authors should justify their choice of supervised vs. unsupervised machine learning here or in the methods section.

Dear Reviewer, we have incorporated the use of supervised machine learning in our study, and have detailed this in Discussion sections.

Supervised machine learning was selected since our goal was to predict and validate genes based on known biological patterns and labels.

Previous studies employing machine learning algorithms in genetics have successfully highlighted key genes associated with disease. Therefore, we applied supervised machine learning techniques, as they effectively enabled the accurate identification of key genes in animals that were affected by BRD.

- Line 252: The authors should justify their choice of using Fischer and Invorm methods for meta-gene identification here or in the method section.

Dear Reviewer, Also, by using *Fisher* and *Invorm*, our approach leads to more meaningful and robust meta-gene identification.

- Line 258-314: This section needs major revisions. There are several instances where the authors reference the role of a gene relative to systems completely unrelated to cattle, BRD, or respiratory disease (e.g, line 264 liver cancer, line 266 cancer cell lines, 273 bowel disease and breast cancer, line 298 Epstein-Barr virus transcytosis, line 299 chagas disease, line 313 endometriosis, line 314 autoimmune disease, etc.). The authors should revise this section and focus on discussing and interpreting their findings within the context of bovine respiratory disease complex since that is the model they used.

Dear Reviewer, we have removed all sentence as suggested.

- Line 277 – 278: Please clarify the relationship between regulating immunological functions and how that specifically “effectively protects” cells against oxidative damage.

Dear Reviewer, the immune system plays a key role in defending the body against pathogens, maintaining cellular homeostasis, protection against oxidative stress. Regulating immunological can help mitigate oxidative damage in controlled inflammation, antioxidant defense, cell signaling and repair, and removal of damaged cells.

- Line 299-301: Could the authors please clarify what important function Epstein-Barr virus transcytosis has in triggering inflammatory genes during BRD. Moreover, could the authors clarify that same information for Chagas disease and mitophagy as well.

Dear Reviewer, we have removed this sentence.

- Line 315-316: Could the authors please be more specific and clarify what genetic and non-genetic factors are relevant to BRD and provide more context for why the authors believe that there should be some genes that regulate the disease process.

Dear Reviewer, *BRD* is affected by genetic and non-genetic factors. Genetic factors include inherited traits such as immune response, and stress resilience that can dispose animals to susceptibility or resistance to the disease. Non-genetic factors involve environmental and management-related conditions such as transportation stress, weaning, weather changes, and overcrowding. Considering that *BRD* is a highly important disease in cattle, there should be some genes that may have considerable expression in the disease process.

Line 317-319: Could the authors clarify why the ML algorithms were suitable for these predictions.

Dear Reviewer, Previous studies employing machine learning algorithms in genetics have successfully highlighted key genes associated with disease. Therefore, we applied supervised machine learning techniques, as they effectively enabled the accurate identification of key genes in animals that were affected by BRD.

- Line 320: That authors should be cautious not to over interpret results, stating that some animals were highly susceptible to BRD when some of the studies analyzed were challenge studies where disease was intentionally induced in animals is misleading to readers.

Dear Reviewer, some of the studies included in this analysis were challenge studies, where BRD was experimentally induced. Therefore, we approached studies on the innate susceptibility of animals to BRD with careful consideration.

- Line 325-326: Please clarify the relevance to BRD.

Dear Reviewer, we have revised the sentence for improved clarity.

- Line 326: Mastitis disease is redundant wording.

Dear Reviewer, we have removed Mastitis disease.

- Line 326-327: This sentence is disruptive to the flow of the discussion and does not provide any meaningful contextualization or aid in the interpretation of the results of the authors study, consider removing.

Dear Reviewer, we have revised the sentence for improved clarity. STAT2 plays a critical role in immune responses and acts as a TF that regulates the expression of numerous genes involved in host defense mechanisms during BRD (65, 66).

- Line 329-330: Please elaborate on this statement. As written it is not clear why NLRC5 can be considered a novel BRD gene.

Dear Reviewer, we have removed the sentence for improved clarity.

- Line 334-335: Please elaborate on the relevance of this to BRD.

Dear Reviewer, we have revised the sentence for improved clarity. The IL15RA was discovered as a potential marker for genetic resistance to infection in animals.

- Line 337: Consider connecting this back to BRD.

Dear Reviewer, we have revised the sentence for improved clarity. ALPK1 is involved in triggering the activation of the inflammatory NF-κB signaling pathway. Additionally, it plays a vital role in the pathogenesis of lung cancer.

- Line 338-339: Please elaborate on the relevance of this to BRD.

Dear Reviewer, we have removed the sentence for improved clarity.

- Line 340: Please be more specific.

Dear Reviewer, we have revised the sentence for improved clarity. Through bioinformatics analysis of RNA-sequencing data, we successfully identified key genes associated with BRD. Additionally, we developed a diagnostic model using a supervised machine learning algorithm to predict potential molecular markers for BRD. This study contributed significantly understanding and identification of molecular markers related to BRD.

**Conclusions:**

- The authors do a good job not overstating most of their findings in the conclusion and highlighting the need for additional research for biomarker discovery.
- Line 346 – 348: Please reword to improve readability. The authors only analyzed RNA-seq and therefore only identified genes that may be of interest. The authors can discuss the relevance of gene clustering with regards to molecular functions immune cell functions, but should be cautious not to over interpret the findings here.

Dear Reviewer, the integrative system biology analysis conducted in the current research demonstrated several important metabolic processes and signaling pathways involved in BRD. Notably, key target genes were identified and used to construct regulatory networks with TFs. By utilizing a supervised ML algorithm in combination with gene clustering approaches, this study provides comprehensive insights into potentially significant genes, immune system components, and molecular functions associated with BRD. These findings not only enhance our understanding of the underlying biological mechanisms but also establish a valuable foundation for future research aimed at the clinical diagnosis, identification of disease biomarkers, and discovery of novel therapeutic targets.
